# Multidimensional transcriptomics based to illuminate the mechanisms of taurine metabolism in immune resistance of pancreatic cancer

**DOI:** 10.3389/fimmu.2025.1567805

**Published:** 2025-03-31

**Authors:** Zongshuai Qin, Guixiang Huang, Jian Xu, Lujuan Pan, Chaojun Lan, Yuhuan Yang, Yixia Yin, Yueqiu Qin

**Affiliations:** ^1^ The First Affiliated Hospital of Jinan University, Jinan University, Guangzhou, Guangdong, China; ^2^ Department of Gastroenterology, Affiliated Hospital of Youjiang Medical University for Nationalities, Baise, Guangxi, China; ^3^ Graduate School, Youjiang Medical University for Nationalities, Baise, Guangxi, China; ^4^ Guangxi Clinical Medical Research Center for Hepatobiliary Diseases, Affiliated Hospital of Youjiang Medical University for Nationalities, Baise, Guangxi, China

**Keywords:** pancreatic cancer, taurine, spatial transcriptomics, immune checkpoint inhibitors, CAFs, immunotherapy

## Abstract

Pancreatic cancer, a highly malignant tumor of the digestive system, is characterized by a tumor microenvironment with a high degree of immunosuppression. This immunosuppressive property poses significant challenges, as it hampers the effective infiltration of immune cells and impairs their ability to exert cytotoxic effects. The metabolic process of taurine has emerged as a crucial factor in modulating the functions and activities of immune cells. Intervening in taurine metabolism holds the potential to reshape the tumor immune microenvironment, thereby enhancing the ability of immune cells to recognize and eliminate tumor cells. To explore the potential therapeutic relationship between taurine metabolism disorders and pancreatic cancer immunotherapy, we employed multiple software packages, including “Seurat”, “DoubletFinder”, “Harmony”, “GSVA”, and “CellChat” to analyze single-cell data and spatial transcriptomic data of pancreatic cancer. In the present study, four distinct tumor cell subsets, namely RPS4Y1+ tumor cells, LYZ+ tumor cells, CPE+ tumor cells, and MKI67+ tumor cells, were identified for the first time. The CNV score and taurine metabolism score highlighted the significant role of RPS4Y1+ tumor cells within the immunosuppressive microenvironment of pancreatic cancer. Through cell-communication analysis, the crosstalk among fibroblasts, CD8+ T cells, and RPS4Y1+ tumor cells was identified, offering novel insights into immunotherapy strategies, which was strengthened by the co-localization analysis of spatial transcriptomics. Furthermore, by conducting a combined analysis of survival data, we identified LY6D as a potential therapeutic target. Through co-culture experiments with fibroblasts, we uncovered the underlying mechanism of LY6D in regulating taurine metabolism imbalance within the immunosuppressive microenvironment of pancreatic cancer. The establishment of the “taurine-immune crosstalk” criteria in this study effectively paves the way for pancreatic cancer immunotherapy. In conclusion, the current research underscores the significance of taurine metabolism in the immunosuppressive microenvironment of pancreatic cancer. Targeting taurine metabolism may represent a crucial approach for reversing the “stiff-cancer” characteristics of pancreatic cancer.

## 1 Introduction

Pancreatic cancer, a highly invasive malignancy within the digestive system, exhibits an exceedingly dire prognosis. In accordance with the most recent cancer statistics, the 5-year survival rate of pancreatic cancer patients has persistently fluctuated within the single-digit range, which indisputably poses a grave threat to human life and health (1). Although chemotherapy and radiotherapy can, to a certain extent, impede the progression of the tumor, owing to the distinctive biological characteristics of pancreatic cancer, encompassing its intricate tumor microenvironment, the high invasiveness of cancer cells, and their drug resistance, the therapeutic outcomes frequently fall short of expectations (2, 3). Pancreatic cancer is renowned as the “hard cancer”. Its tumor microenvironment is replete with a substantial quantity of extracellular matrix and cancer-associated fibroblasts (CAFs), which jointly form a dense physical barrier, significantly impeding the infiltration of immune cells into the tumor tissue (1). Simultaneously, immunosuppressive cells in the pancreatic cancer microenvironment, such as regulatory T cells (Tregs) and myeloid-derived suppressor cells (MDSCs), amass in large numbers. They incessantly secrete a diverse array of immunosuppressive factors, such as transforming growth factor-β (TGF-β) and interleukin-10 (IL-10), further suppressing the activity and function of immune cells, rendering it arduous for immunotherapy to fully exert its efficacious anti-tumor effect (4–6).

Immune checkpoint inhibitors reactivate the anti-tumor activity of immune cells by obstructing the interaction between these molecules. However, as aforementioned, in the intricate tumor microenvironment of pancreatic cancer, the efficacy of immune checkpoint inhibitors is severely constrained (7). In light of the abundant fibroblasts in pancreatic cancer, researchers have been continuously delving into novel treatment strategies in recent years. Some studies have endeavored to reshape the tumor microenvironment by targeting CAFs, thereby enhancing the efficacy of immunotherapy (2, 8). For instance, some studies have utilized small-molecule inhibitors to block cytokines secreted by CAFs, such as platelet-derived growth factor (PDGF), to inhibit the activation and proliferation of CAFs, thereby reducing the production of extracellular matrix and ameliorating the infiltration environment of immune cells (9). Nevertheless, these methods are still in the clinical trial phase, and no breakthrough progress has been achieved. They still confront numerous challenges in terms of safety, efficacy, and large-scale application (10).

Taurine, a sulfur-containing amino acid, its metabolic pathway principally encompasses two pivotal processes: synthesis and transportation (11). In terms of synthesis, taurine is predominantly synthesized from cysteine through a series of intricate enzymatic reactions. Intracellular taurine is implicated in numerous crucial physiological processes, such as regulating cell osmotic pressure, maintaining the stability of the intracellular milieu, and exerting an antioxidant stress-alleviating role (11). In tumor cells, aberrations in the taurine metabolic pathway are intimately associated with the occurrence, development, and metastasis of tumors (12). Studies have revealed that some tumor cells highly express taurine transporter (TauT) and uptake a substantial amount of taurine to meet their requirements for rapid proliferation and survival (13). Meanwhile, taurine can also influence the immune escape process of tumors by modulating the functions of immune cells in the tumor microenvironment. In recent years, the pivotal role of taurine metabolism in cancer immunotherapy has gradually garnered extensive attention from the academic community. There exists a close and intricate relationship between taurine metabolism and the tumor immune microenvironment (14, 15). On the one hand, taurine can markedly modulate the functions and activities of immune cells. For example, taurine can enhance the cytotoxic activity of natural killer cells (NK cells) and cytotoxic T lymphocytes (CTLs) and promote their recognition and elimination of tumor cells (16). Research has demonstrated that taurine can enhance the expression of cytotoxicity-related molecules by regulating the signal transduction pathways in NK cells and CTL cells, thereby augmenting their ability to kill tumor cells (17). On the other hand, taurine can also regulate the functions of immunosuppressive cells, effectively diminishing the immunosuppressive effects of Tregs and MDSCs. Taurine can inhibit the proliferation and activation of Tregs and MDSCs and reduce the level of immunosuppressive factors they secrete, thus ameliorating the tumor immune microenvironment. By intervening in the taurine metabolic pathway, such as administering taurine supplements or inhibiting the activity of taurine synthase, the growth and metastasis of tumors can be significantly modified (18).

With the rapid advancement of biotechnology, spatial transcriptomics and single-cell transcriptomics technologies have furnished unprecedentedly powerful tools for the in-depth exploration of tumor biology and cancer immunotherapy. Through single-cell transcriptome analysis, we can profoundly understand the heterogeneity of tumor cells, immune cells, stromal cells, etc. in pancreatic cancer, as well as the complex interaction relationships among them. Spatial transcriptomics technology further compensates for the limitations of single-cell transcriptomics technology. It can precisely localize and analyze gene expression while preserving the spatial architecture of tissues. In the investigation of the relationship between taurine metabolism and immune resistance in pancreatic cancer, the integration of spatial transcriptomics and single-cell transcriptomics technologies is of great significance. The combination of these technologies aids in comprehensively analyzing the cell-cell interactions and molecular regulatory networks in the tumor microenvironment from a spatiotemporal perspective, providing a more robust theoretical foundation for the development of novel treatment strategies. Therefore, this paper elucidates the molecular mechanism of taurine metabolism in the immune resistance of pancreatic cancer by comprehensively applying these technologies and methods, furnishing a solid theoretical basis for the development of innovative treatment strategies, and thereby bringing new therapeutic hope to pancreatic cancer patients.

## 2 Methods

### 2.1 Identification and annotation of cell subsets

Three cases of primary pancreatic ductal adenocarcinoma were collected from the previous study (GSE197177) by Lv et al., and the data were preprocessed using the “Seurat” package. Within the cell analysis workflow, cell clustering is accomplished through the utilization of the “FindNeighbors” and “FindClusters” functions. In the cell annotation phase, by referring to the cell subset markers within the CellMarker database, integrating with the typical markers validated in prior studies, and subsequently employing the automatic annotation function of “SingleR”, the annotation is meticulously refined to guarantee accurate cell classification. To identify the differentially expressed genes (DEGs) of diverse cell subsets, the “FindAllMarkers” function is activated. The parameters min.pct and min.diff.pct are set at 0.25, and the logFC threshold is also set at 0.25 to screen out genes with significant differential expression. Given the high degree of heterogeneity exhibited by tumor cells, they are re-clustered. Subsequently, the specific marker genes of tumor cells are utilized to annotate the tumor cell subsets, thereby enabling an in-depth analysis of the characteristics of tumor cells.

### 2.2 Identification of PC cells

The computation of CNV levels is executed through the use of the Copykat (version 1.1.0) and inferCNV (version 1.12.0) software. Among these, Copykat is employed to conduct copy number microarray analysis of aneuploid tumors, with the aim of discerning between malignant and non-malignant cell types. Concurrently, the inferCNV software, with NK cells serving as the reference, is utilized to determine whether additional cancer cells display significant chromosomal copy number variations (CNV).

### 2.3 Functional prediction and enrichment analysis

To conduct an in-depth analysis of the DEGs of distinct cell types, the “ClusterProfiler” R package (version 4.6.0) is utilized to perform Gene Ontology (GO) and Kyoto Encyclopedia of Genes and Genomes (KEGG) enrichment analyses. GO enrichment analysis aids in elucidating the roles of genes within cells and comprehending their functions from the perspectives of cellular components, molecular functions, and biological processes. KEGG enrichment analysis focuses on metabolic and signaling pathways, enabling an understanding of the key physiological pathways in which genes are implicated, thus facilitating a profound exploration of the biological implications underlying DEGs. The Gene Set Enrichment Analysis (GSEA) software (version 4.1.0) is carefully downloaded from the GSEA website (http://software.broadinstitute.org/gsea/msigdb). Simultaneously, the pathway gene sets are retrieved from the MSIGDB database (https://www.gseamsigdb.org/) and are systematically analyzed using the GSEA software. The “DESeq2” R package is selected, and the threshold is set as | logFC| > 2 and p-value < 0.05.

### 2.4 Differentiation characteristics of tumor subsets

The “Monocle” R package (version 2.24.0) is employed to meticulously analyze the pseudo-time trajectory of tumor cells. During this process, the UMAP method is utilized for dimensionality reduction. Post-dimensionality reduction, the “PLOT_CELL_TRACTURE” function is employed for visualization. Different cell subsets are precisely sorted according to the pseudo-time sequence, and the genes that exhibit synchronous changes along the pseudo-time trajectory are identified. A pseudo-time heatmap is utilized to depict the changes in these genes, with the heatmap visually representing the high and low levels of gene expression through different colors. Additionally, the “Slingshot” R package (version 2.6.0) is selected. The “getlineage” function assists in constructing the framework of the cell development lineage, while the “getCurves” function focuses on calculating the expression levels of different lineages within the fitting time.

### 2.5 Analysis of the potential communication network between tumor cells and other cells

The “CellChat” package is utilized to infer the intricate interactions between cells and construct a regulatory network based on the ligand-receptor level. The “netVisual DiffInteraction” function is employed to illustrate the disparities in the communication intensity between cells. These disparities in communication intensity reflect the degree of activity in signal exchange between different cell pairs. The “IdentifyCommunicationPatterns” function is used to estimate the number of communication patterns. The significance threshold is set at 0.05 (p-value), and only when the p-value is lower than this threshold is the corresponding result deemed statistically significant. Circular plots, bubble plots, and violin plots are utilized. The circular plot comprehensively presents the incoming and outgoing signals of all cells.

### 2.6 Identification of transcription factor modules

The “pySCENIC” R package within Python (version 3.7) is employed to conduct single-cell regulatory network inference and clustering analysis. Initially, the “GRNBoost” algorithm is utilized to identify the potential target genes of each transcription factor. Subsequently, DNA motif analysis is carried out to identify the genes that directly interact with transcription factors from the potential target genes identified by “GRNBoost”. Finally, “AUCell” is utilized to score the activity of regulators in cells. By comprehensively considering factors such as gene expression levels, a quantitative score is assigned to the activity of each regulator in cells. The human gene motif ranking data involved in this study are sourced from https://resources.aertslab.org/cistarget/.

### 2.7 Establishment of the “taurine-immune disruption” criterion

The expression values of patient characteristic genes are precisely extracted from the TCGA_PC cohort. On this foundation, univariate Cox regression analysis is conducted to preliminarily explore the association between each characteristic gene and PC prognosis. Subsequently, LASSO Cox regression analysis (employing the glmnet tool, version 4.1-6) is utilized to impose specific constraints on the regression coefficients, enabling the effective identification and management of multicollinearity. Multivariate Cox regression analysis is employed to calculate the risk coefficients of each gene. Based on these coefficients, a prognostic risk-scoring model is constructed. In accordance with the calculated median risk score, patients are clearly classified into high-risk and low-risk groups. Subsequently, the Kaplan–Meier formula within the “Survival” R package (version 3.3-1) is utilized to conduct survival analysis on the TCGA-PC cohort. The “ggsurviplot” function is used to visualize the survival curve. The “Time ROC” R package is used to plot the curve of the area under the ROC curve (AUC) as it changes over time. By observing the variations in AUC at different time points, the prediction ability of the model at different stages can be understood. The survival rate calibration plot, ROC curve, and C-index are plotted to comprehensively evaluate the prediction ability of the nomogram.

### 2.8 Identification of immunotherapy-benefiting populations based on taurine metabolism crosstalk

The “CIBERSORT” R package (version 0.1.0) is utilized to conduct an in-depth evaluation of 22 types of immune cells within the tumor immune microenvironment of different patient groups. The “ESTIMATE”, “CIBERSHOT” and “Xcell” algorithms are also employed to quantitatively assess the stromal and immune components within the tumor microenvironment. Subsequent to the analysis of each algorithm, the obtained scores are integrated and visualized with the assistance of the “ggpubr” R package. Spearman correlation analysis is utilized to explore the relationship between the risk score and the genes constituting the risk score. The Tumor Immune Dysfunction and Exclusion (TIDE) algorithm (accessible at http://tide.dfci.harvard.edu/) is used to evaluate the efficacy of immune checkpoint inhibitors (ICI) in the treatment of cancer patients.

### 2.9 Spatial transcriptome analysis

Two pieces of spatial transcriptomics slice data of pancreatic cancer, GSM6505134 and GSM6505135, were downloaded from the GEO database. Comprehensive Spatial Transcriptomics data analysis is carried out with the aid of the R package “stCancer”. This encompasses the pre-quality control (QC) step to ensure the accuracy of subsequent analyses. Subsequent clustering analysis is performed. Additionally, gene expression analysis is conducted to investigate the spatial distribution of LY6D. To accurately assess the spatial distribution of the cell subsets identified from the single-cell cohort, the R package “CellTrek” is utilized to merge and co-dimension the ST and scRNA-seq expression matrices. Subsequently, the “scoloc” function is employed to summarize the co-localization patterns between different cell subsets, and the “Kullback-Leibler divergence (KLD)” is used to measure the intensity of cell co-expression. It is important to note that the higher the KLD value, the stronger the co-localization degree of cell subsets. Moreover, the R package “CARD” is utilized for deconvolution analysis in this study. When differentiating between malignant regions (Mal), boundaries (Bdy), and non-malignant regions (nMal), the R package “Cottrazm” is selected for region identification, and the cell enrichment scores of different regions are evaluated according to the previously reported deconvolution method. Finally, the “Wilcoxon test” is employed to determine whether the differences in cell enrichment in different regions are statistically significant.

### 2.10 Cell culture

In this study, all the pancreatic cancer cell lines employed are sourced from the American Type Culture Collection. Simultaneously, human pancreatic tumor fibroblasts (immortalized) are procured from EYKITs Biotechnology Company. The RPMI-1640 medium is supplemented with 10% fetal bovine serum and 1% streptomycin/penicillin to ensure an appropriate cell culture environment. The culture temperature is set at 37°C, and the carbon dioxide concentration within the culture environment is regulated to 5%. In addition, the co-culture model was simulated using a specialized Transwell nested model. Tumor cells were placed in the upper layer, and fibroblasts were placed in the lower layer to mimic the interaction pattern of cytokines.

### 2.11 Cell transfection

In the experiment, to downregulate the expression of the LY6D gene, the sh-LY6D knockdown plasmid from GenePharma Company in Suzhou, China, is selected. During the transfection procedure, the steps outlined in the product manual of Lipofectamine 3000RNAiMAX, manufactured by Invitrogen Company in the United States, are strictly adhered to. The cells to be treated are seeded in a 6-well plate. When the cell confluence reaches 50%, the transfection process commences. In each transfection process, Lipofectamine 3000RNAiMAX (Invitrogen, USA) is consistently used as the transfection reagent to ensure the consistency and stability of the transfection experimental conditions.

### 2.12 Cell functional experiments

To explore the effects of sh-LY6D transfection on cells, the cell viability and invasion ability of pancreatic cancer cells are assayed. For the assessment of cell viability, the CCK-8 assay is utilized. The specific procedure is as follows: Initially, the cells are prepared into a suspension and seeded into a 96-well plate at a density of 5×10³ cells per well. Subsequently, they are incubated within the culture environment for 24 hours. Then, 10 μL of CCK-8 labeling agent is added to each well. After the addition of the labeling agent, the 96-well plate is placed in an environment at 37°C, protected from light, and incubated for an additional 2 hours. Subsequently, the absorbance values at 450 nm are continuously measured. First, the starved cells are treated with Matrigel. Following this treatment, the cell suspension is added to the upper chamber containing Costar, and the medium containing serum is added to the lower chamber. The assembly is then placed in the incubator for 48 hours. After the culture period, the cells are fixed with 4% paraformaldehyde and subsequently stained with crystal violet.

### 2.13 Data analysis

In this study, all statistical analyses of publicly available data were conducted using R version 4.2.3 and Python version 3.9. Specifically, for the statistical analysis of high-dimensional data, we relied on the built-in functions of R packages, which provided a robust framework for handling and interpreting complex datasets. The quantification of statistical differences in the experimental outcomes was carried out via the built-in programs of Graphpad. These programs are well-established in the scientific community for their accuracy and reliability in performing statistical calculations. Regarding the survival curves, each was subjected to a log-rank test and analyzed using the Kaplan-Meier method. This approach is a gold standard in survival analysis, enabling a comprehensive assessment of the survival probabilities over time. For datasets exhibiting non-normal distribution, the Kruskal-Wallis test was routinely employed. This non-parametric test is particularly suitable when the data do not meet the assumptions of normality. Conversely, for normally distributed data, either one-way analysis of variance (ANOVA) or a two-tailed t-test was utilized to evaluate and conclude the statistical significance. These parametric tests are powerful tools for detecting differences between groups under the condition of normal distribution. Throughout this study, a p-value of less than 0.05 was considered indicative of a statistically significant difference, denoted by an asterisk (*). This convention is widely adopted in scientific research to delineate significant findings from those that may occur by chance.

## 3 Results

### 3.1 Identification of principal cell constituents and segregation of malignant cells within the PC microenvironment

Prior to advancing our investigations, we utilized DoubletFinder (version 2.0.3) to eliminate doublets. Simultaneously, we implemented stringent quality control measures as delineated in the methodological framework to exclude substandard cells. Furthermore, we employed Harmony (version 0.1.0) in conjunction with the top 30 principal component analysis (PCA) components derived from the samples to ameliorate batch effects. Consequently, 22,124 cells were categorized into distinct clusters using the UMAP methodology at a resolution of 1.0, classifying them into monocytes, epithelial cells, CD4+ T cells, CD8+ T cells, NK cells, macrophages, B cells, dendritic cells (DCs), mast cells, endothelial cells, fibroblasts, and plasma cells (**Supplementary Figure S1A**). These clusters were delineated based on the unique gene expression profiles of each cellular group, with specific marker genes for each cluster detailed in **Supplementary Figure S1B**. To precisely identify malignant cells within the microenvironment to enhance immunotherapeutic strategies, we applied the Copykat algorithm for meticulous malignant cell identification, distinguishing between benign and malignant cell clusters based on differential malignant cell scores (**Supplementary Figure S1C**).

**Figure 1:**
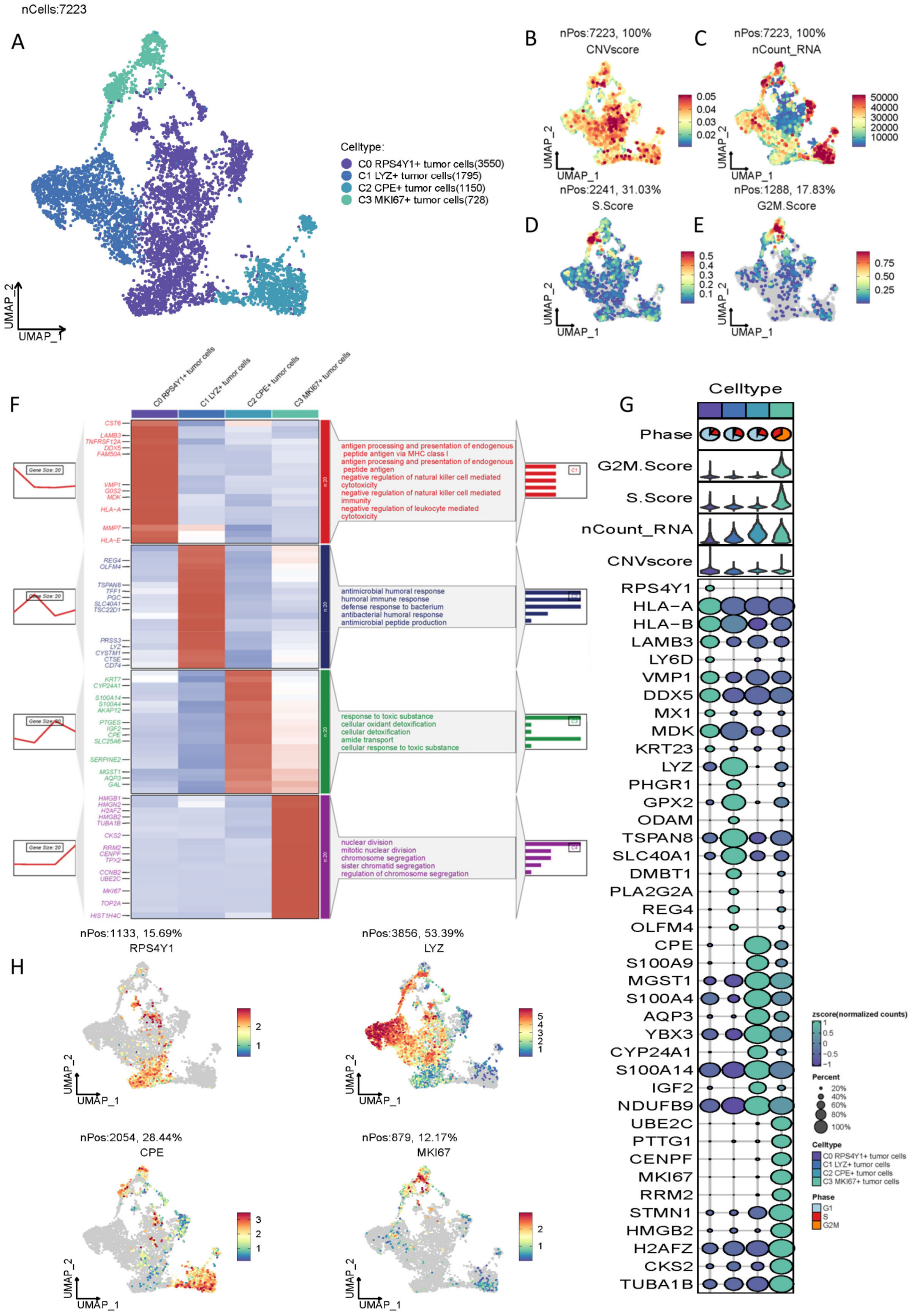
Characteristics of malignant cell types in PC tumors identified by scRNA-seq. **(A)** UMAP plot visualizing 4 tumor cell clusters. **(B-E)** Heatmaps showing the characteristics of CNVscore, nCount_RNA, S.score, and G2M.score in different tumor cell clusters. **(F)** Marker clustering heatmap and pathway enrichment analysis of the four malignant cell clusters. **(G)** Bubble plot showing the differences among markers of different clusters and the correlations of CNVscore, nCount_RNA, S.score, and G2M.score characteristics. **(H)** UMAP heatmap of the most significant markers of the cell clusters.

Subsequent to the successful isolation of malignant epithelial cells, we performed re-clustering of the malignant cells, resulting in the classification of 7,223 pancreatic cancer malignant cells into four distinct clusters: C0 RPS4Y1+ tumor cells (3,550), C1 LYZ+ tumor cells (1,795), C2 CPE+ tumor cells (1,150), and C3 MKi67+ tumor cells (728) (**Figures 1A–C**). These clusters were evaluated based on CNV score, nCount RNA, S.Score, and G2M.Score (**Figures 1B–E**). The findings revealed that C0 RPS4Y1+ tumor cells exhibited the highest malignancy score, whereas S.Score and G2M.Score were comparatively lower for this cluster. To further elucidate the functional attributes of these subpopulations, we conducted Gene Set Enrichment Analysis (GSEA) on the four subpopulations. The results demonstrated that the C0 subpopulation, characterized by the highest malignancy, was predominantly enriched in functions associated with antigen processing and presentation of endogenous peptide antigen via MHC class, negative regulation of natural killer cell-mediated cytotoxicity, and negative regulation of leukocyte-mediated cytotoxicity (**Figure 1F**), suggesting that the C0 cell cluster is primarily implicated in immune suppression. Other cell clusters were mainly enriched in functions such as response to toxic substances. The highly expressed markers of these cell clusters are illustrated in (**Figure 1G**), with the most representative markers for each subpopulation depicted in (**Figure 1H**).

### 3.2 Proliferative dynamics and taurine-mediated interactions among tumor subpopulations

**Figure 2:**
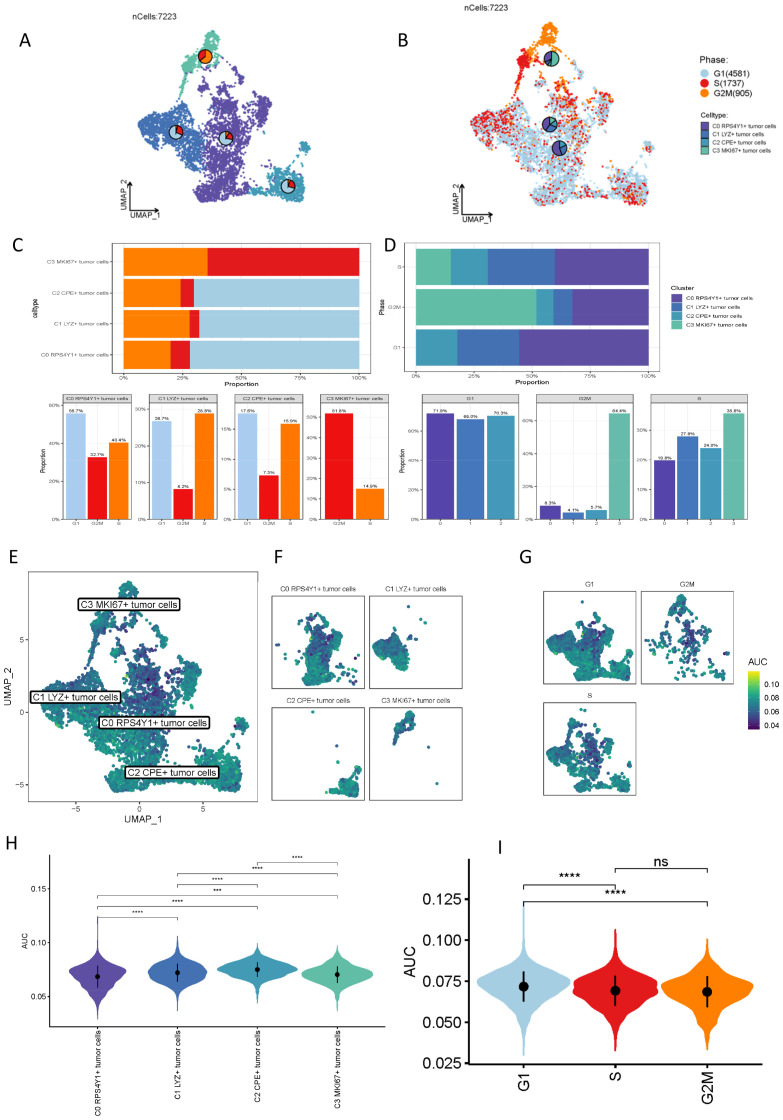
Proportion distribution of different malignant cell clusters and comparison of taurine metabolism characteristics. **(A, B)** Cell cycle changes of the four clusters of malignant tumor cells. **(C, D)** Proportions of different malignant cell subsets in different cell cycles. **(E, F)** UMAP plots of the taurine metabolic pathway in different malignant cell subsets. **(G)** Representation of different cell cycles of cells with different taurine metabolism. **(H, I)** Violin plots showing the taurine metabolism status in different cell subsets and different cell cycles. ns, not significant, * p < 0.05, *** p < 0.001, **** p < 0.0001.

As previously indicated, the four malignant tumor subpopulations exhibit potential functional significance. To more precisely delineate the distinctions among these cellular entities, we subsequently scored the four clusters based on G2M, G1, and S phases (**Figures 2A, B**). The data illustrate that different tumor cell subpopulations occupy various mitotic phases. To more effectively represent these distinctions, we conducted a statistical analysis of these cell clusters, with the results presented in (**Figures 2C, D** **).** We conducted statistics respectively according to the number of cells in each subgroup of different cell cycles and the proportion of different cell subgroups in different cell cycles. The C0 cell cluster is predominantly in the G1 phase, indicative of active RNA and protein synthesis, coupled with heightened energy metabolism. Other cell clusters are primarily in the G2M and S phases, suggesting a greater involvement in DNA replication. Our focus then shifted to the differential taurine-mediated interactions among these cell subpopulations (**Figures 2E–G**). Recent studies have demonstrated that taurine metabolism interacts with tumor immunity, modulating immune cells and the tumor microenvironment, while tumor immunity reciprocally influences taurine metabolism, offering novel insights for tumor treatment. Through AUCell scoring of different cell subpopulations, we identified that the C0 cell cluster possesses the lowest taurine score, whereas the C2 cell cluster exhibits the highest, aligning with our prior research findings (**Figure 2H**). Given that taurine metabolism can enhance T cell proliferation and activation, augmenting their cytotoxic efficacy to more effectively identify and eradicate tumor cells, thereby bolstering the body’s anti-tumor immune response, the C0 cell cluster, characterized by the highest malignancy, suggests that within the pancreatic cancer microenvironment, this cluster suppresses the activity of the taurine metabolic pathway, potentially serving as a pivotal target for future pancreatic cancer immunotherapy. This aspect is further emphasized in the G2M scoring (**Figure 2I**).

### 3.3 Temporal differentiation attributes of tumor subpopulations

**Figure 3:**
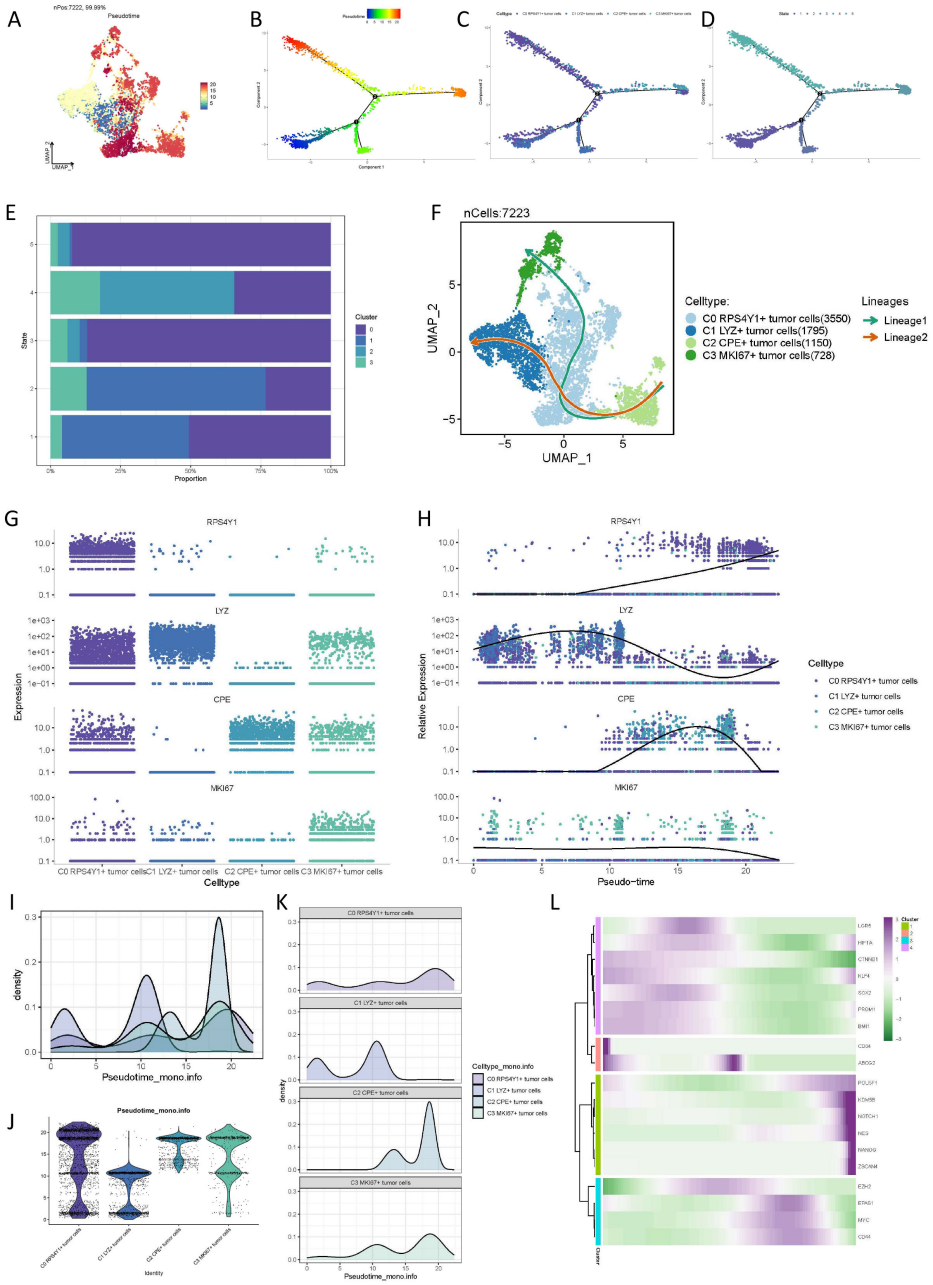
Pseudo-time trajectory differentiation analysis of tumor subsets. **(A-D)** Results of Monocle showing the differentiation states of different cell subsets. **(E)** Proportions of different malignant cell subsets in different cell differentiation states. **(F)** Slingshot evolution trajectory analysis revealing the evolution laws of different cell cycles. **(I-K)** Pseudo-time scores quantifying the trajectories of different cells. **(L)** Heatmap based on pseudo-time trajectory analysis to provide the top markers of each pancreatic cancer cell subtype.

To investigate the differentiation characteristics of distinct pancreatic cancer cell subtypes, we employed Monocle2 to analyze the differentiation of the four subtypes. In comparison to other subtypes, the C0 cell subpopulation, distinguished by the highest CNV and lowest taurine index, occupies the midpoint and endpoint of cell differentiation, indicating its propensity to transition into the terminal stage of cellular development. By determining the state of cells in chronological order using Monocle2, the results showed that the C0 cell cluster was distributed in various states, but it was mainly in the intermediate state and the terminal state, that is, state 3 and state 5. (**Figures 3A–D**). Further analysis utilizing slingshot elucidated the temporal sequence of the four cell subpopulations, revealing that C0 RPS4Y1+ tumor cells are situated at the midpoint of the trajectory and participate in two simulated evolutionary pathways, culminating in C1 LYZ+ tumor cells and C3 MKi67+ tumor cells, respectively (**Figures 3E, F**). As the pseudotime trajectory progresses, the expression dynamics of malignant cell subtype markers (RPS4Y1, LYZ, MKi67, and CPE) are depicted in (**Figures 3G, H**). Additionally, we quantified the state of the four cell subtypes using Pseudotime scoring (**Figures 3I–K**). Moreover, based on pseudotime trajectory analysis, the top markers of each tumor subtype are displayed in the heatmap (**Figure 3L**).

### 3.4 Intercellular communication within the pancreatic cancer tumor microenvironment

**Figure 4:**
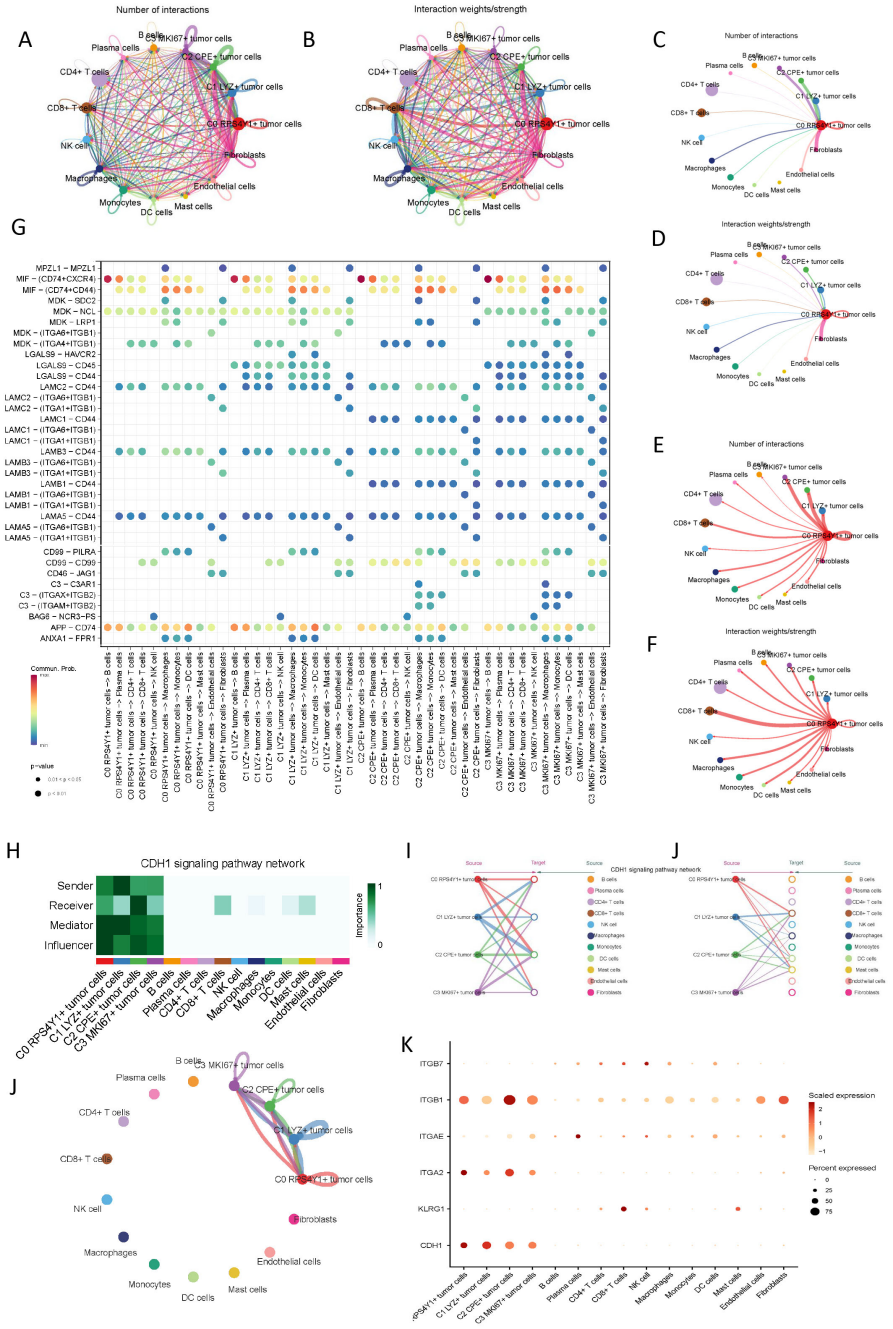
Microenvironment cell interaction patterns of different malignant cells in PC. **(A, B)** Circ plots showing the number and intensity of interactions among all cells. **(C)** Number of interactions between C0 RPS4Y1+ tumor cells as receivers and other cells. **(D)** Interaction intensity between C0 RPS4Y1+ tumor cells as receivers and other cells. **(E)** Number of interactions between C0 RPS4Y1+ tumor cells as senders and other cells. **(F)** Interaction intensity between C0 RPS4Y1+ tumor cells as senders and other cells. **(G)** Bubble plot showing the communication network between tumor cells and different cells in the microenvironment. **(H-K)** Communication patterns of the CDH1 signaling pathway among tumor cells of different subsets.

Cells within the tumor microenvironment do not exist in isolation. To more comprehensively decipher the intercellular communication dynamics of the four tumor subpopulations previously mentioned, we utilized CellChat to explore cellular interactions, aiming to infer the interplay between different tumor cells and other cellular entities, as illustrated in (**Figures 4A, B**). Given the unique taurine imbalance characteristics of C0 RPS4Y1+ tumor cells within the pancreatic cancer tumor microenvironment, we further scrutinized the intercellular communication status of this subpopulation. We observed cellular crosstalk centered around C0 RPS4Y1+ tumor cells. Fibroblasts emerged as the predominant source of cellular signals to C0 RPS4Y1+ tumor cells, suggesting that fibroblasts may play a pivotal role in mediating PC taurine imbalance and tumor immunity (**Figures 4C–F**). Conversely, C0 RPS4Y1+ tumor cells transmitted more signals to CD8+ T cells. Subsequently, we examined the ligand-receptor pairs of these signals, noting that ligands such as MIF, MDK, and APP are highly expressed on C0 RPS4Y1+ tumor cells and are instrumental in mediating communication with other tumor microenvironments (**Figure 4G**). Additionally, we focused on the signaling pathways between tumor cells. Within the CDH1-dominated signaling pathway, the four tumor subpopulation cells interacted with each other, with C0 RPS4Y1+ tumor cells primarily being influenced, hinting at the evolutionary dynamics and crosstalk among tumor cells (**Figures 4H–K**).

### 3.5 Transcription factor network analysis of malignant cell subsets

**Figure 5:**
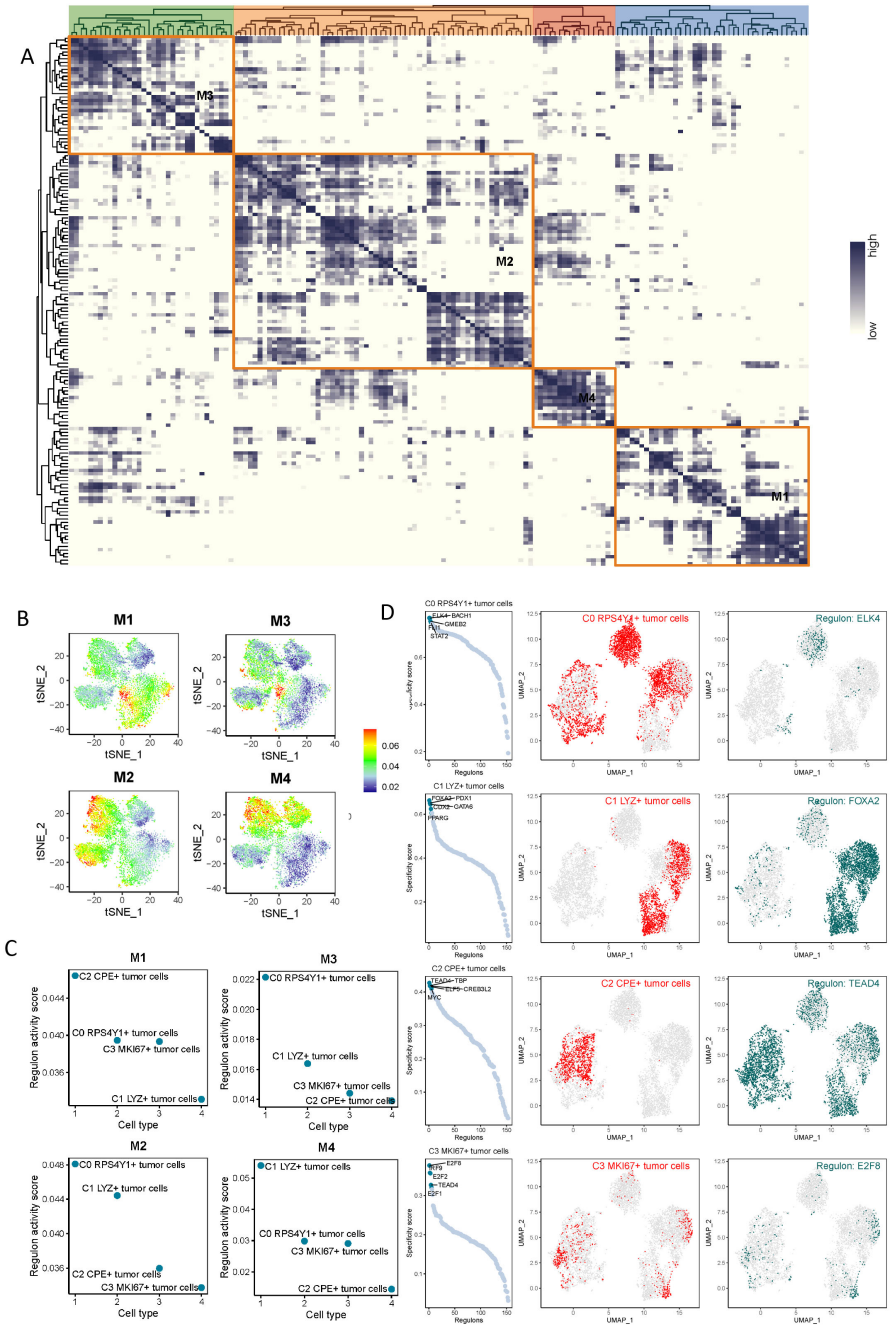
Transcription factor regulatory network analysis of tumor cell subsets. **(A, B)** Based on the connectivity-specific index (CSI) matrix, the regulator modules and representative transcription factors of tumor cell subtypes were determined. **(C, D)** Regulators in pancreatic cancer tumor cell subtypes were ranked based on the regulator-specific score (RSS) and the most significant transcription factors were selected.

Based on the above-mentioned research, we conducted a SCENIC analysis to identify the core transcription factors (TFs) that could be recognized in tumor cell subtypes. The gene regulatory networks of all sub-clusters of malignant cells were reconstructed using pySCENIC, and these regulators were divided into four specific modules (M1-4) (**Figure 5A**). According to the activity scores, it can be seen that the four modules mainly corresponded to different cell subsets. C0 RPS4Y1+ tumor cells mainly corresponded to the M3 and M2 modules, C1 LYZ+ tumor cells mainly corresponded to the M4 module, C2 CPE+ tumor cells mainly corresponded to the M1 module, and the specificity of C3 MKl67+ tumor cells was not very significant (**Figure 5A**). FLK4, BACH1, and GMEB2 were important regulators of C0 RPS4Y1+ tumor cells. The regulators in C1 LYZ+ tumor cells were mainly FOXA2, PDX1, GATA6, and PPARG. TEAD4, TBP, MYC, and ELF5 constituted the transcription factor regulatory network of C2 CPE+ tumor cells. The transcription factor network of C3 MKl67+ tumor cells was mainly composed of E2F1, E2F2, etc. (**Figure 5B**). In addition, the UMAP plot more clearly presented the transcription factor networks of different subsets after dimensionality reduction, indicating that the functions of these TFs were unique to each subtype (**Figure 5C**). Moreover, each module occupied a specific region, and these modules showed a complementary pattern. There were specific transcription factors in these networks, namely ELK4, FOXA2, TEAD4, and E2F8 (**Figure 5D**).

### 3.6 Establishment of the specific scoring criteria for the taurine metabolic pathway in PC

**Figure 6:**
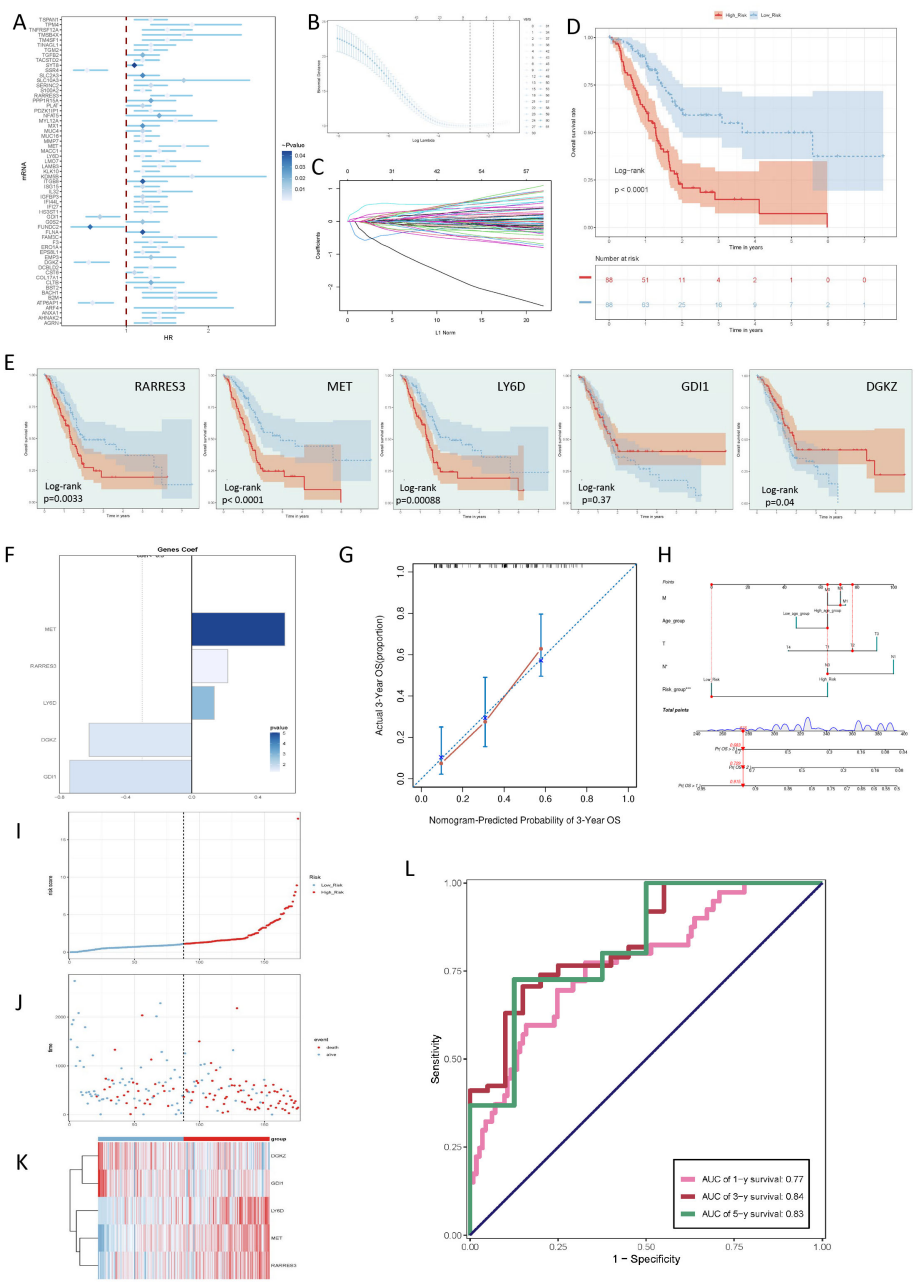
Screening of key genes for tumor prognosis and construction of taurine metabolism crosstalk criteria. **(A-C)** Univariate Cox regression analysis of prognosis-related gene risks and LASSO regression analysis for modeling. **(D)** Risk prognosis of populations with different taurine metabolism crosstalk criteria. **(E)** KM survival curve status of genes for criterion construction. **(F)** Coefficient values of the 5 genes selected by the LASSO Cox regression model. **(G, H)** Calibration curves of the nomogram for predicting PC patients. **(I)** Curve showing the risk scores of different taurine metabolism crosstalk criteria. **(J)** Scatter plot showing the survival status of survival/death events in the two groups over time. **(K)** Heatmap of the differential expression of genes composing the taurine metabolism crosstalk criteria. **(L)** AUC values for predicting the 1st, 3rd, and 5th-year outcomes in the TCGA cohort.

To analyze whether C0 RPS4Y1+ tumor cells could affect the anti-tumor immune effect, we first developed a risk scoring system based on the top 100 marker genes of C0 RPS4Y1+ tumor cells. These genes were examined using univariate Cox regression, and further screened using LASSO regression (**Figure 6A**). The results showed that a total of 5 genes were significantly associated with the prognosis of patients (**Figures 6B, C**). We named this discrimination criterion the “taurine-immunity” criterion. The current scoring method could well distinguish the prognosis of patients, which strengthened the significance of taurine imbalance in the population (**Figure 6D**). Based on the risk score, with the median as the cut-off point, patients were divided into different risk groups. The current discrimination criterion could well illustrate the differences among patients (**Figures 6D–F**). At the same time, we used a nomogram to more accurately evaluate this criterion (**Figures 6G–K**). To evaluate the prognostic efficacy of the risk score, the area under the ROC curve was used to judge its prediction of different time-related outcomes. The results showed that the area under the ROC curve (AUC) values for the 1st, 3rd, and 5th years were 0.77, 0.84, and 0.83, respectively (**Figure 6L**).

### 3.7 Identification of immunotherapy-benefiting populations based on taurine metabolism crosstalk

**Figure 7:**
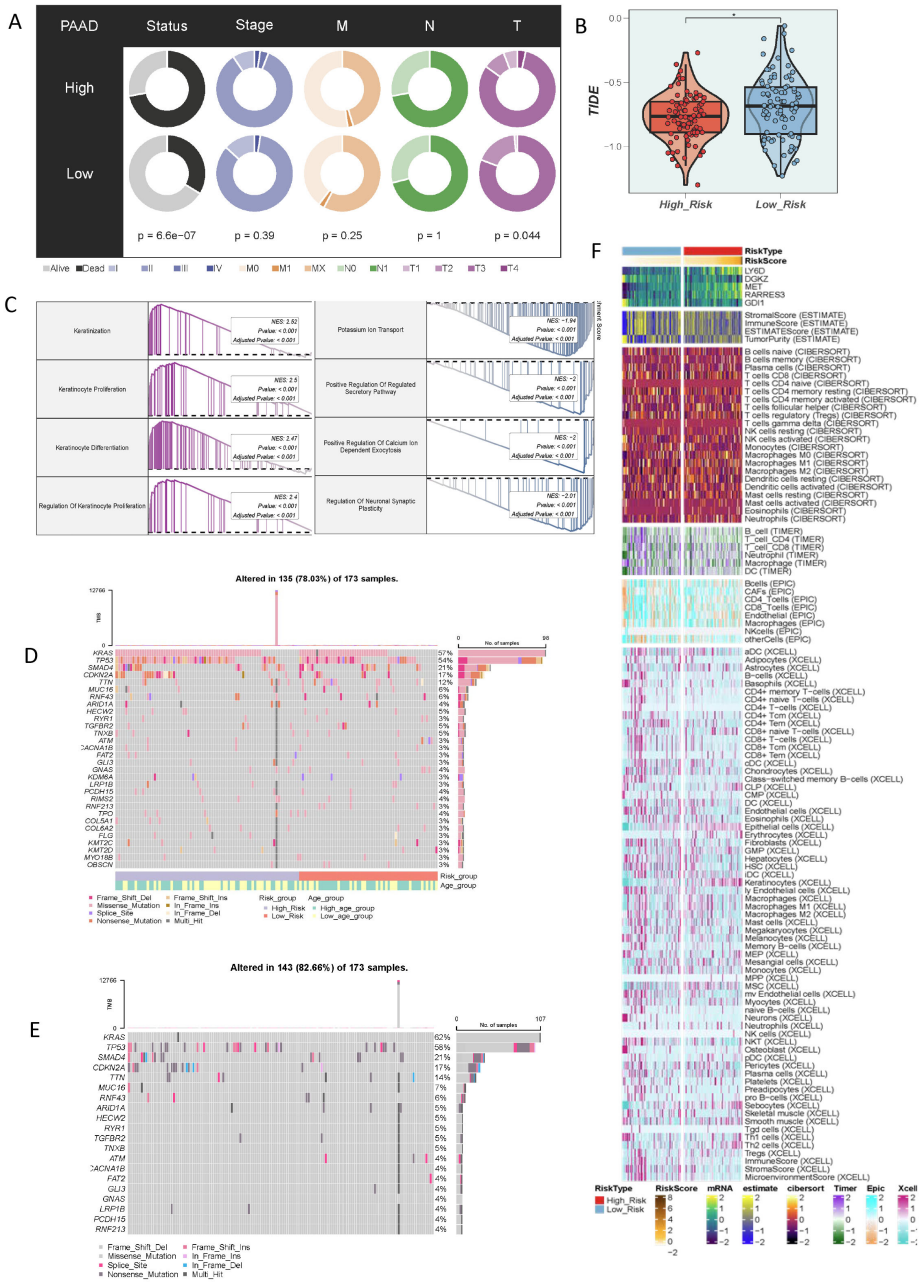
Characteristics of the immune microenvironment of populations divided by different taurine metabolism crosstalk criteria. **(A)** Correlations of clinical parameters in populations with different criteria. **(B)** TIDE score status of populations with different taurine metabolism crosstalk criteria. **(C)** Enrichment results of differential populations. **(D, E)** Gene mutation status of populations with different taurine metabolism crosstalk criteria. **(F)** Heatmap showing the ESTIMATE, CIBERSORT, and Xcell results of PC. * p < 0.05.

In addition, we evaluated the relationship between the current score and different clinical characteristics. The results showed that the current criterion could significantly distinguish the disease states of different patients and was related to the T-stage (**Figure 7A**). Moreover, we predicted the tumor immunotherapy response using the TIDE score. The results showed that the TIDE score was low in the high-risk population, suggesting a disorder of immune escape in the high-risk population (**Figure 7B**). Then, we performed a GSEA analysis on the differences among different populations. The results were mainly enriched in pathways such as Keratinization and Keratinocyte Proliferation (**Figure 7C**). The most exciting evidence of the current study was that, through the evaluation of the immune microenvironment, we found that the high-risk population was accompanied by more infiltration of immunosuppressive cells, including fibroblasts, which strengthened the conclusions of the above-mentioned research (**Figure 7F**). In addition, we studied the gene mutation information of different populations. The results showed that the high-risk population was accompanied by more gene mutations in KRAS, TP53, and SMAD4 (**Figures 7D, E**).

### 3.8 LY6D as a potential link between taurine metabolism and immune resistance in pancreatic cancer

**Figure 8:**
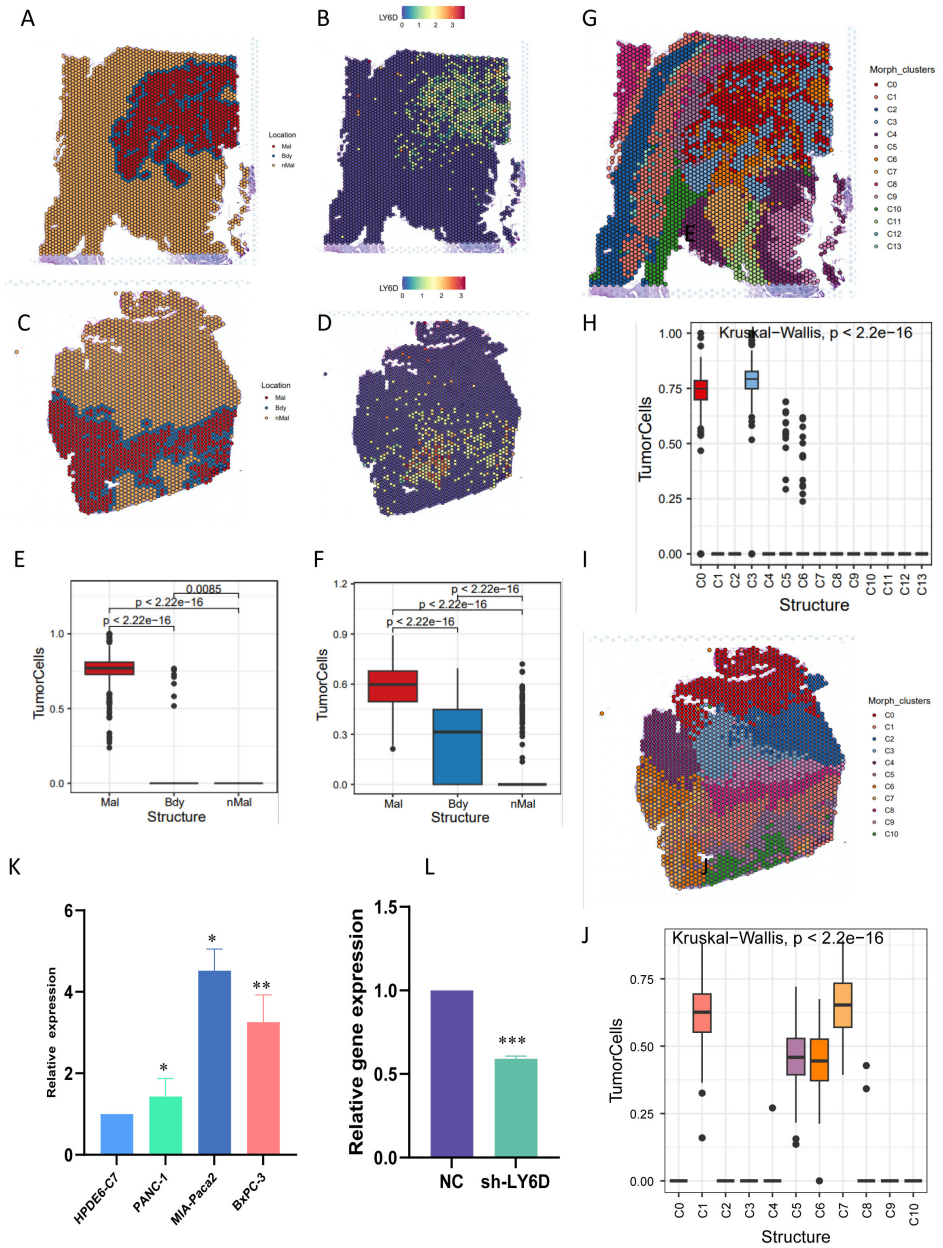
Validation of LY6D expression by combining spatial transcriptomics and RT-PCR. **(A-D)** Tumor boundary algorithm and LY6D gene expression in two groups of spatial transcriptome sections. **(E, F)** Bar graphs representing the LY6D expression differences in the tumor boundary segmentation of the two groups of sections. **(G-J)** Spatial expression of LY6D after mapping by the Morphological adjusted Cluster algorithm. **(K)** LY6D expression in common pancreatic cancer cell lines. **(L)** Gene expression after plasmid-mediated knockdown of LY6D. ns not significant, * p < 0.05, ** p < 0.01, *** p < 0.001.

**Figure 9:**
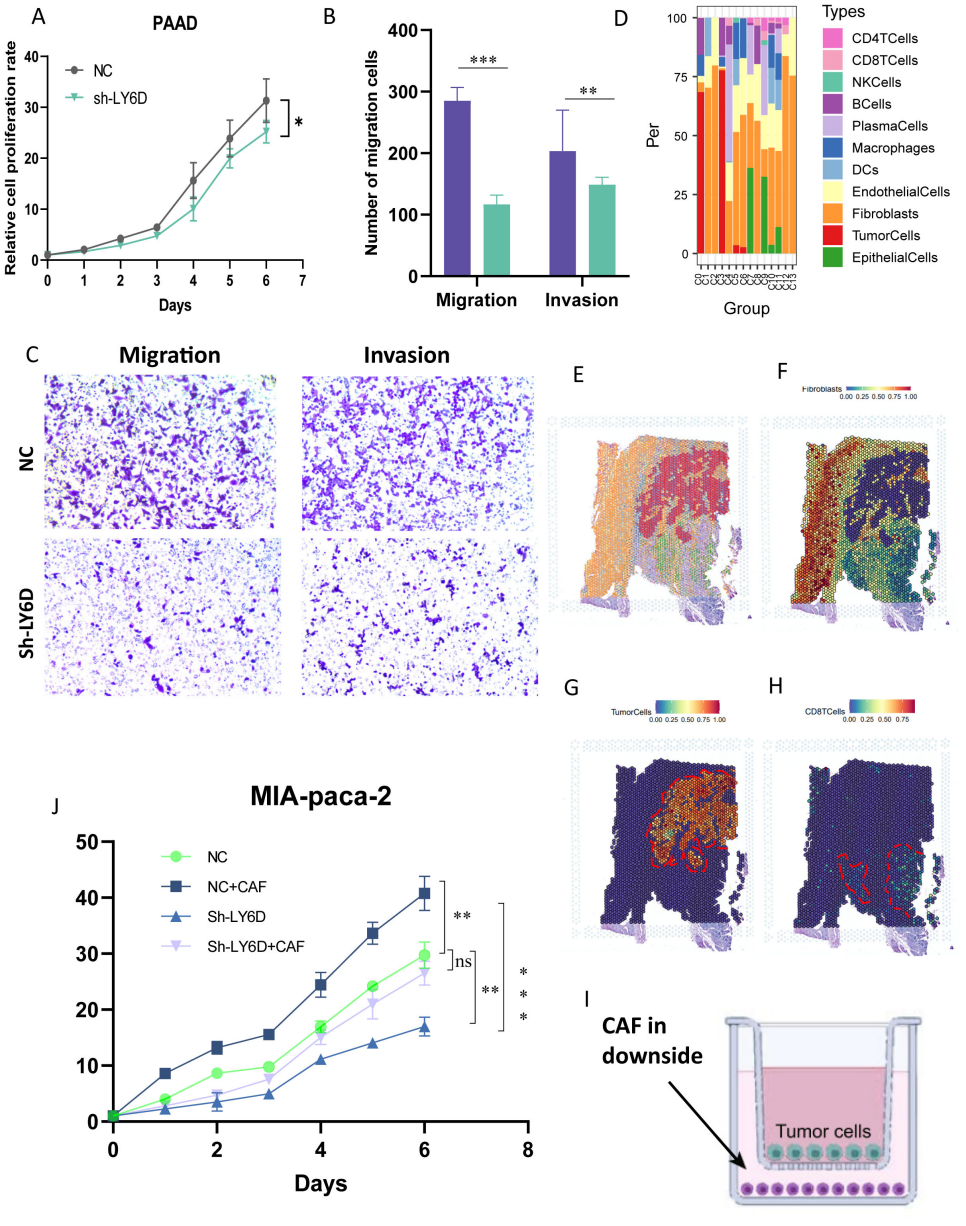
LY6D expressed by malignant tumor cells attracts fibroblasts to induce malignant progression of pancreatic cancer. **(A)** CCK8 assay showing that the growth of pancreatic cancer cells decreased after down-regulating LY6D. **(B, C)** The invasion and migration abilities of pancreatic cancer cells decreased after down-regulating LY6D. **(D)** Proportion of cell distribution in different clusters of spatial transcriptomics. **(E-H)** Mapping of tumor fibroblasts and malignant tumor cells in spatial transcriptomics. **(I, J)** Cell interaction experiments in the co-culture system revealing that LY6D is an intermediate in inducing the malignant progression of pancreatic cancer by fibroblasts. * p < 0.05, ** p < 0.01, *** p < 0.001.

Combined with the literature review and the current research foundation, LY6D was selected as a candidate marker linking taurine metabolism and immune resistance in pancreatic cancer. In addition, through the analysis of immune infiltration of LY6D in different cohorts of pancreatic cancer, it can be observed that LY6D is mainly associated with fibroblast infiltration and has little relationship with other immune cells. Crucially, LY6D shows a negative correlation with CD8+ T cells and cytotoxic lymphocytes, indicating that it may mediate the immune suppression of these two cell types (**Supplementary Figure S3**). Combining the literature review and the current research basis, LY6D was selected as a candidate marker linking taurine metabolism and immune resistance in pancreatic cancer. Therefore, we first performed spatial localization on the spatial transcriptome sections of pancreatic cancer using the tumor boundary algorithm (**Figures 8A–D**). The results showed that cells with high LY6D expression were concentrated in the tumor core of pancreatic cancer, and there was a statistically significant difference (**Figures 8E, F**). In addition, we performed a localization analysis on the spatial spots using Morphological adjusted cluster. The results showed that the sections were divided into 13 and 10 co-localization clusters respectively, and tumor cells were only located in specific clusters (**Figures 8G–J**). This indicated that LY6D was specifically located in the tumor core region of pancreatic cancer tissue, strengthening our previous speculation that it could be a tumor marker (**Figures 8G–J**). We experimentally verified the expression level of LY6D in common pancreatic cancer cells. The results showed that LY6D was highly expressed in pancreatic cancer cells such as PANC-1, MIA-Paca2, and BxPC-3, and was most highly expressed in MIA-Paca2 (**Figures 8K, L**). In further research, we down-regulated the expression level of LY6D in MIA-Paca2 cells. The results showed that the decrease in LY6D expression was accompanied by a decrease in cell viability and invasion and migration abilities (**Figures 9A–C**). As mentioned above, C0 RPS4Y1+ tumor cells had the most communication with fibroblasts and CD8 T cells. We hypothesized that LY6D might be an important factor promoting the transformation of the “hard cancer” characteristics of pancreatic cancer, which made it impossible for T cells to attack the tumor core region, resulting in immunosuppression. Then, the single-cell samples were stratified according to the proportion of C0 RPS4Y1^+^ tumor cells to create a “high C0 score” group and a “low C0 score” group. We compared the functions of CD8^+^ T cells between these two groups in order to better determine whether the presence of RPS4Y1^+^ tumor cells is associated with impaired T cell responses. We observed that cells with a high C0 score tended to have a negative correlation with T cells and a positive correlation with fibroblasts, which further reinforces our conclusion (**Supplementary Figure S4**). Therefore, we performed a spatial analysis of the cell distribution in each section of the spatial transcriptome. The results showed that in different co-localization regions, the distribution of tumor cells and fibroblasts was accompanied by a decrease in T cells, which verified our previous conclusion (**Figures 9D–H**). Then we performed co-culture of fibroblasts and tumor cells. The results showed that the viability of PC cells co-cultured with CAF could be significantly enhanced, but down-regulating LY6D reduced this enhancement, however, in the Ly6D down-regulation group, the viability of PAAD cells was significantly enhanced after co-culturing with CAFs, indicating the non-negligible role of LY6D in the communication between PC cells and CAF cells (**Figures 9I–J**).

## 4 Discussion

In recent years, cancer immunotherapy has emerged as a focal point in the realm of cancer research, garnering substantial attention. Owing to its distinctive treatment paradigm and mode of action, it has bestowed novel prospects of remission upon cancer patients (19–21). The tumor microenvironment of pancreatic cancer is intricate and exhibits a pronounced immunosuppressive phenotype, rendering it arduous for immune cells to exert their normal anti-tumor functions. Consequently, tumor cells can elude the body’s immune surveillance and assaults (22–24). Taurine metabolism, an essential physiological process within organisms, bears a close and intricate relationship to the tumor immune microenvironment (25). In the context of the tumor immune microenvironment, aberrant taurine metabolism can enable tumor cells to circumvent the body’s immune surveillance by modulating the functions of immune cells (26, 27). The elevated uptake of taurine by tumor cells is a prevalent phenomenon. This process may afford favorable conditions for the proliferation and survival of tumor cells by regulating the intracellular redox equilibrium, maintaining osmotic homeostasis, and influencing signal transduction cascades (25). Taurine may also partake in the intracellular signal transduction process, activating signal pathways associated with cell proliferation and fostering the division and growth of tumor cells (28).

During the in-depth exploration of the pancreatic cancer microenvironment, four distinct cell populations were successfully identified and isolated, namely C0 RPS4Y1+ tumor cells, C1 LYZ+ tumor cells, C2 CPE+ tumor cells, and C3 MKl67+ tumor cells. From the vantage point of fundamental cellular biological characteristics, these four cell populations manifested marked disparities in key indices such as CNV score, nCount RNA, S.Score, and G2M.Score. Among them, C0 RPS4Y1+ tumor cells exhibited the highest malignancy score, while their S.Score and G2M.Score were comparatively low, intimating unique attributes of this cell population within the cell cycle progression. Through GSEA to probe their functional characteristics, it was discerned that the C0 subgroup with the highest malignancy was predominantly enriched in biological functions such as antigen processing and presentation, negative regulation of natural killer cell-mediated cytotoxicity, and negative regulation of leukocyte-mediated cytotoxicity. This strongly implies that the C0 cell cluster plays a pivotal role in the immunosuppressive microenvironment of pancreatic cancer.

It is worthy of note that C0 RPS4Y1+ tumor cells exhibited the unique characteristics of a low taurine score and a high CNV index. Taurine assumes a dual-function role in tumor biology. Under normal physiological circumstances, it plays a crucial part in the activation and functional sustenance of immune cells, effectively augmenting the body’s anti-tumor immune response. Taurine can stimulate the proliferation and activation of T cells, enhance their cytotoxicity, endowing them with the capacity to more efficaciously recognize and eliminate tumor cells. In the tumor microenvironment, particularly in a highly malignant milieu such as that of C0 RPS4Y1+ tumor cells, the taurine metabolic pathway may be inhibited. The C0 cell cluster has the highest degree of malignancy, signifying that within the tumor microenvironment of pancreatic cancer, this group of cells represented by the C0 cell cluster can suppress the activity of the taurine metabolic pathway within the overall microenvironment of pancreatic cancer cells. A high CNV index reflects a substantial augmentation in the genomic instability of C0 RPS4Y1+ tumor cells. This instability is closely intertwined with the malignant progression of tumors, which may prompt tumor cells to acquire additional genetic variations, thereby enhancing their proliferative, invasive, and metastatic capabilities. A low taurine score may augment the ability of tumor cells to evade the body’s immune surveillance, as the promoting effect of taurine on T-cell function is impeded, rendering it easier for tumor cells to elude immune attacks and thus fostering tumor development (29, 30).

Pancreatic cancer is renowned as the “hard cancer” due to the fact that its tumor microenvironment is replete with a profusion of extracellular matrix and CAFs (10, 31). In the investigation of cell-cell communication, it was discovered that C0 RPS4Y1+ tumor cells had the most frequent communication with fibroblasts. Fibroblasts furnished the most potent cell signals to C0 RPS4Y1+ tumor cells. This phenomenon indicates that fibroblasts are likely to be a pivotal link in mediating taurine imbalance and tumor immunity in PC. The intimate communication between fibroblasts and C0 RPS4Y1+ tumor cells may impinge on the “hard cancer” characteristics of pancreatic cancer via multiple pathways. Fibroblasts are capable of secreting a copious amount of extracellular matrix components, such as collagen and fibronectin, which accumulate in large quantities within tumor tissues, forming a dense physical barrier that obstructs the infiltration of immune cells into the tumor tissue (32). The signal communication between C0 RPS4Y1+ tumor cells and fibroblasts may regulate the activation and proliferation of fibroblasts, further propelling the production of extracellular matrix. The interaction between the two may also impact the activity of the taurine metabolic pathway in the tumor microenvironment, exacerbating taurine imbalance and thereby exerting a deleterious effect on the efficacy of tumor immunotherapy (10, 33). In the process of tumor immunity, CD8+ T cells, as pivotal immune killer cells, can specifically recognize and eliminate tumor cells (34). However, owing to the immunosuppressive microenvironment in which C0 RPS4Y1+ tumor cells are situated, the functions of CD8+ T cells may be severely circumscribed. Multiple factors within the tumor microenvironment, such as immunosuppressive factors, immune checkpoint molecules, and regulatory immune cells, act upon CD8+ T cells, inhibiting their activation, proliferation, and cytotoxic functions (35). An in-depth exploration of the relationship between C0 RPS4Y1+ tumor cells and CD8+ T cells is of great significance for elucidating the immune escape mechanism of pancreatic cancer and devising effective immunotherapy strategies. By unravelling the interaction mechanism between the two, it is anticipated to identify key targets to surmount the immunosuppressive microenvironment, thereby enhancing the anti-tumor activity of CD8+ T cells (36).

This study centers on the role of LY6D as a potential linchpin molecule in the nexus between taurine metabolism and immunotherapy. Although there are relatively scarce studies on the specific mechanism of LY6D in this domain at present, preliminary investigations have intimated its potential significant associations. In the tumor microenvironment, LY6D may indirectly influence the tumor immune microenvironment by regulating the expression of key enzymes or transporters in the taurine metabolic pathway. LY6D may modulate the expression of the Taut, thereby affecting the uptake of taurine by tumor cells, altering the intracellular taurine levels in tumor cells, and ultimately influencing the proliferative, survival, and immune escape capabilities of tumor cells (37). Further in-depth research on the mechanism of LY6D in the relationship between taurine metabolism and immunotherapy is anticipated to proffer novel targets and strategies for the treatment of pancreatic cancer (38).

Looking ahead, the future research directions can be primarily centered around the following aspects: Firstly, it is imperative to conduct an in-depth exploration of the precise regulatory mechanism of the taurine metabolic pathway within the tumor microenvironment of pancreatic cancer. This involves identifying the key regulatory nodes and molecules, thereby laying a solid theoretical foundation for the development of therapeutic strategies that target taurine metabolism. By elucidating these intricate regulatory processes, we can gain a more profound understanding of how taurine metabolism impacts pancreatic cancer immune resistance and develop more effective therapeutic interventions. A comprehensive and thorough investigation of the interaction mechanisms between C0 RPS4Y1+ tumor cells and other cells is required. Thirdly, systematic research on LY6D is necessary. Unraveling the role of LY6D in these processes may lead to the development of innovative therapeutic approaches that specifically target this molecule to improve the treatment of pancreatic cancer. Integrating multi-omics technologies, such as proteomics and metabolomics, is essential for comprehensively analyzing the molecular characteristics of the tumor microenvironment of pancreatic cancer. This integration will provide more comprehensive and profound theoretical support for the precise treatment of pancreatic cancer. By combining data from different omics platforms, we can obtain a more holistic view of the molecular landscape of the tumor microenvironment, which is crucial for developing personalized and effective treatment strategies.

## Data Availability

The original contributions presented in the study are included in the article/**Supplementary Material**. Further inquiries can be directed to the corresponding authors.

## Supplementary material

Supplementary Figure 1 Identification of Cell Types and Malignant Cells. **(A)** Two-dimensional UMAP-dimensionality-reduced plot of cell types. **(B)** Key marker genes of different cell types. **(C)** Identification of malignant epithelial cells.

Supplementary Figure 2 Expression of Key Genes in the Taurine Metabolic Pathway in Different Malignant Cell Subsets. **(A)** SLC6A8. **(B)** GOT2. **(C)** NFAT5. **(D)** ANXA5. **(E)** TIMP1. **(F)** PSAP.

Supplementary Figure 3 The relationship between LY6D and immune infiltration. **(A)** The relationship between LY6D and the infiltration of different immune cells in different pancreatic cancer cohorts. **(B)** GSE57495. **(C)** GSE28735. **(D)** GSE85916.

Supplementary Figure 4 The differences in immune cell infiltration and immune factors between the “high C0 score” group and the “low C0 score” group. **(A)** The immune cell infiltration in the “high C0 score” group and the “low C0 score” group. **(B)** The correlation between the immune cell infiltration in the “high C0 score” group and the “low C0 score” group and common immune chemokines.
