# Supplementary figures and images for: Multidimensional transcriptomics based to illuminate the mechanisms of taurine metabolism in immune resistance of pancreatic cancer
Source: Front Immunol. 2025 Mar 31;16:1567805. doi: 10.3389/fimmu.2025.1567805 (PMC11994670; doi:10.3389/fimmu.2025.1567805)

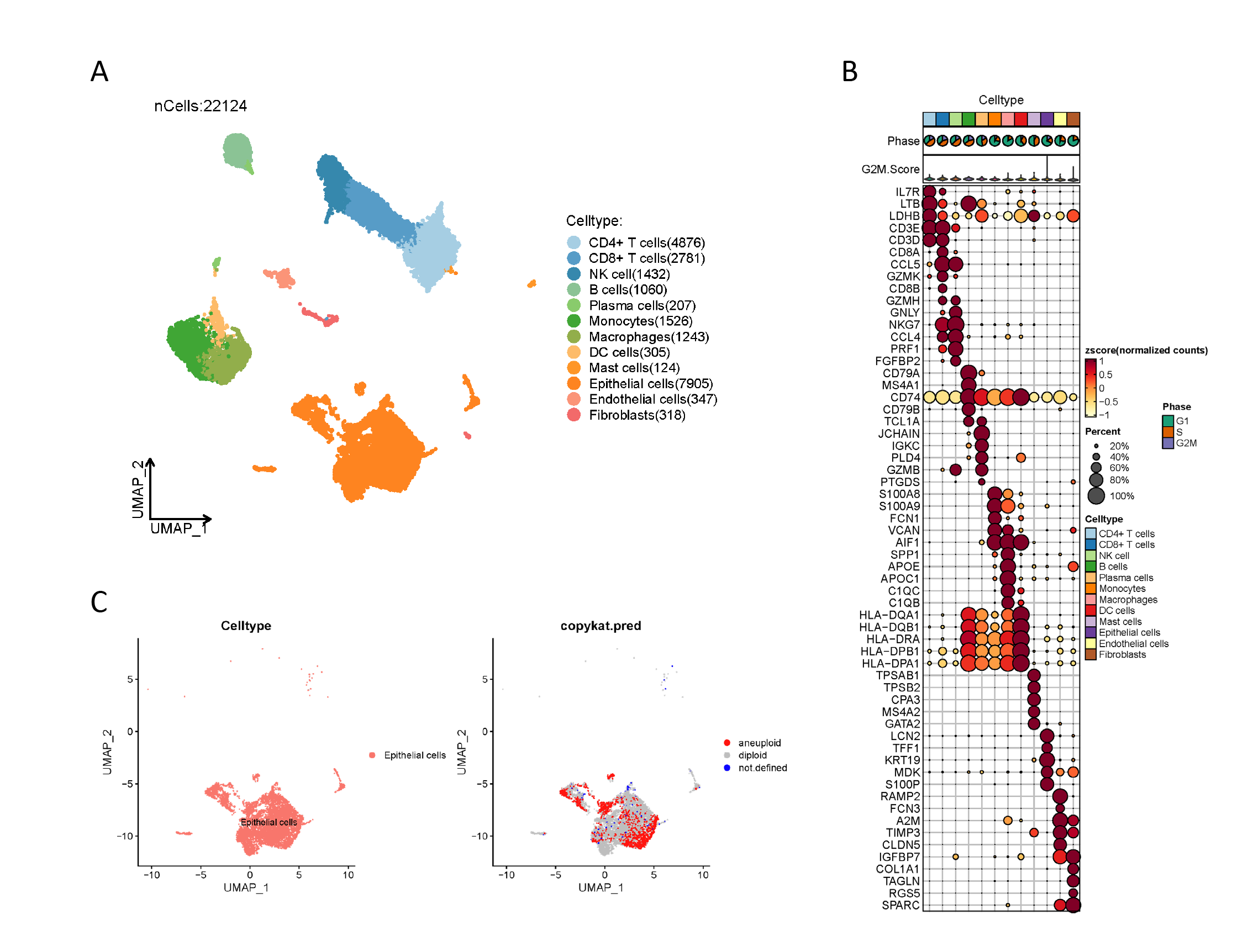

Supplement: Supplementary Figure 1 — Identification of Cell Types and Malignant Cells. (A) Two-dimensional UMAP-dimensionality-reduced plot of cell types. (B) Key marker genes of different cell types. (C) Identification of malignant epithelial cells. [file Image1.tif]

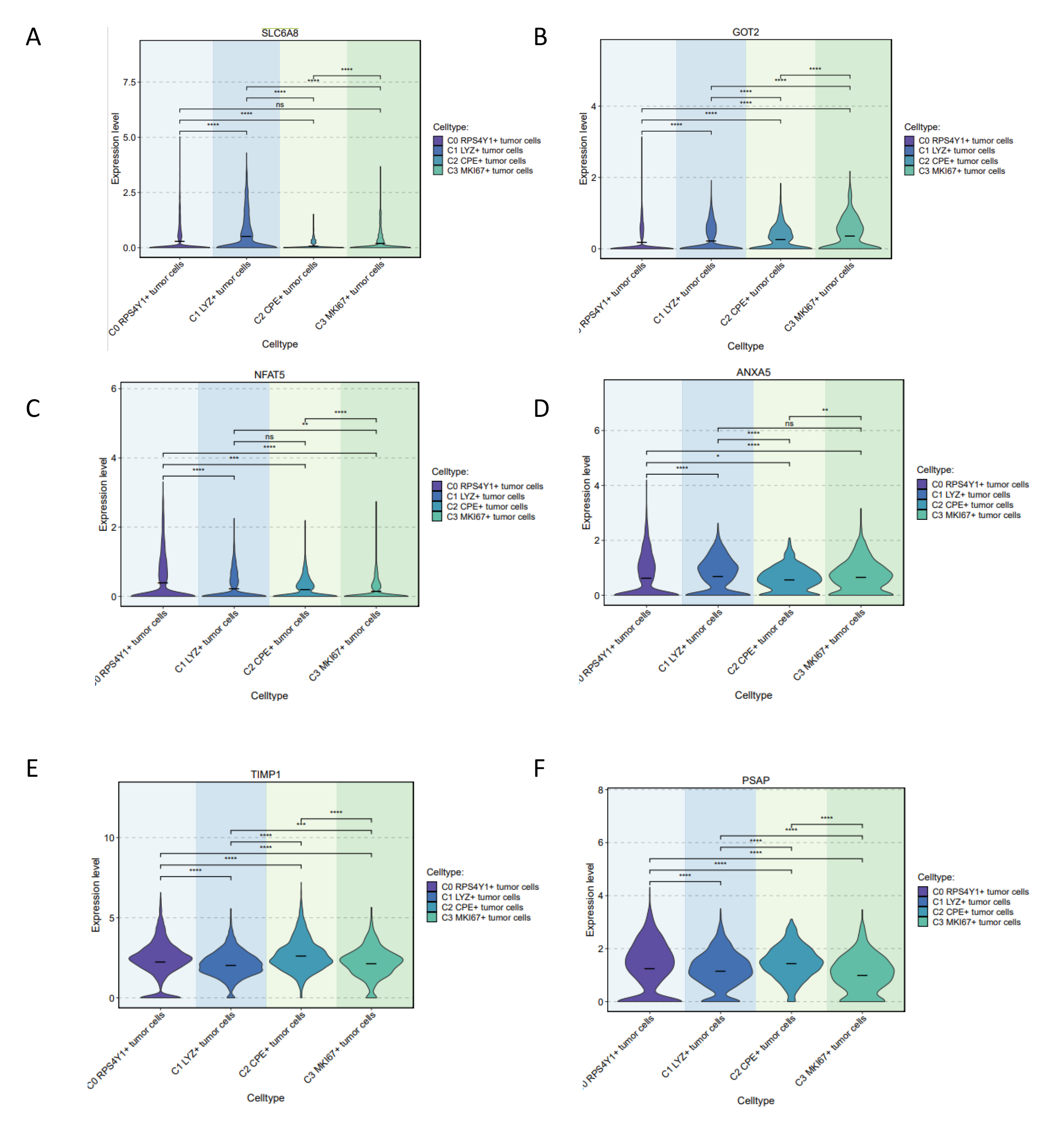

Supplement: Supplementary Figure 2 — Expression of Key Genes in the Taurine Metabolic Pathway in Different Malignant Cell Subsets. (A) SLC6A8. (B) GOT2. (C) NFAT5. (D) ANXA5. (E) TIMP1. (F) PSAP. [file Image2.tif]

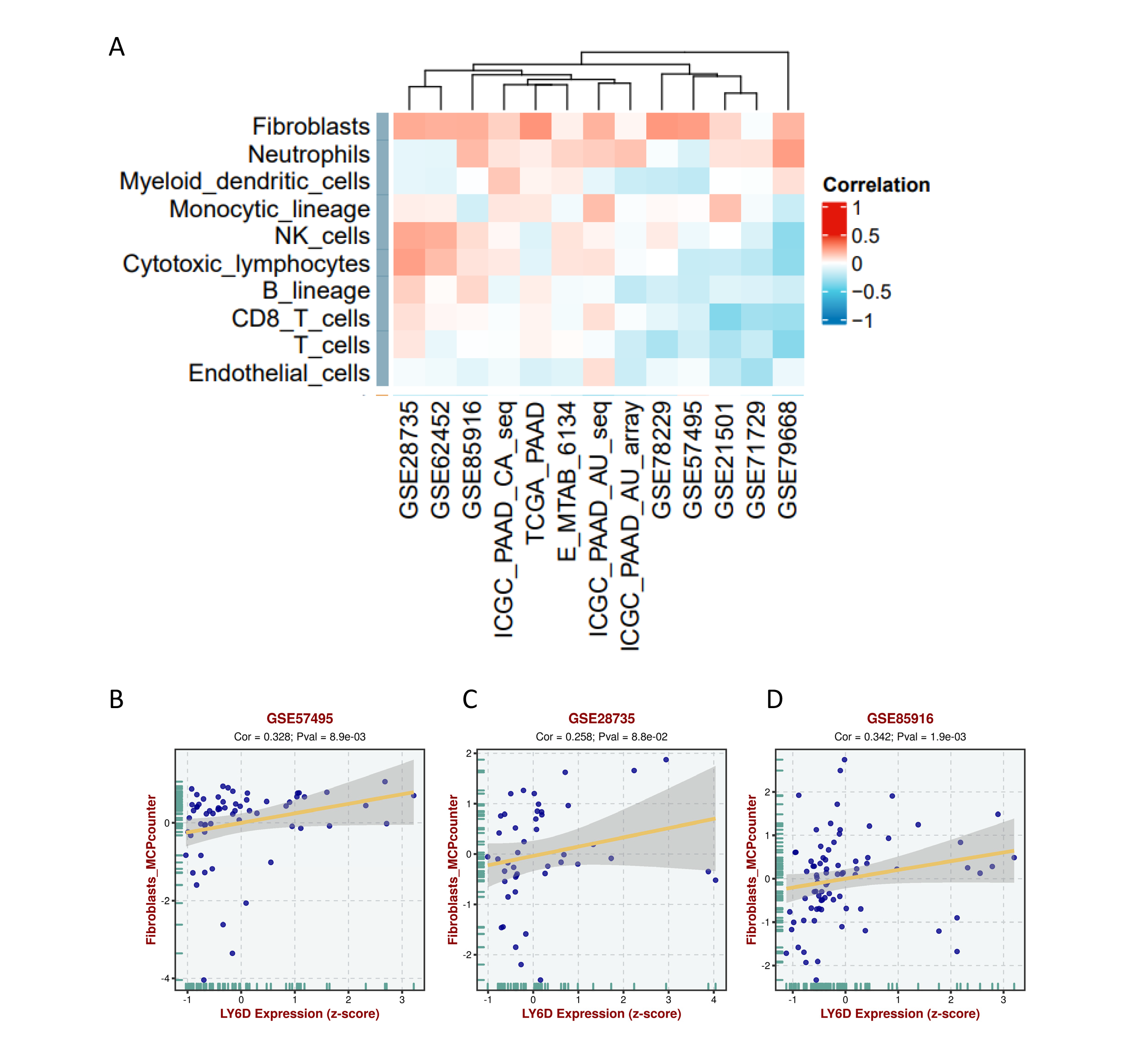

Supplement: Supplementary Figure 3 — The relationship between LY6D and immune infiltration. (A) The relationship between LY6D and the infiltration of different immune cells in different pancreatic cancer cohorts. (B) GSE57495. (C) GSE28735. (D) GSE85916. [file Image3.tif]

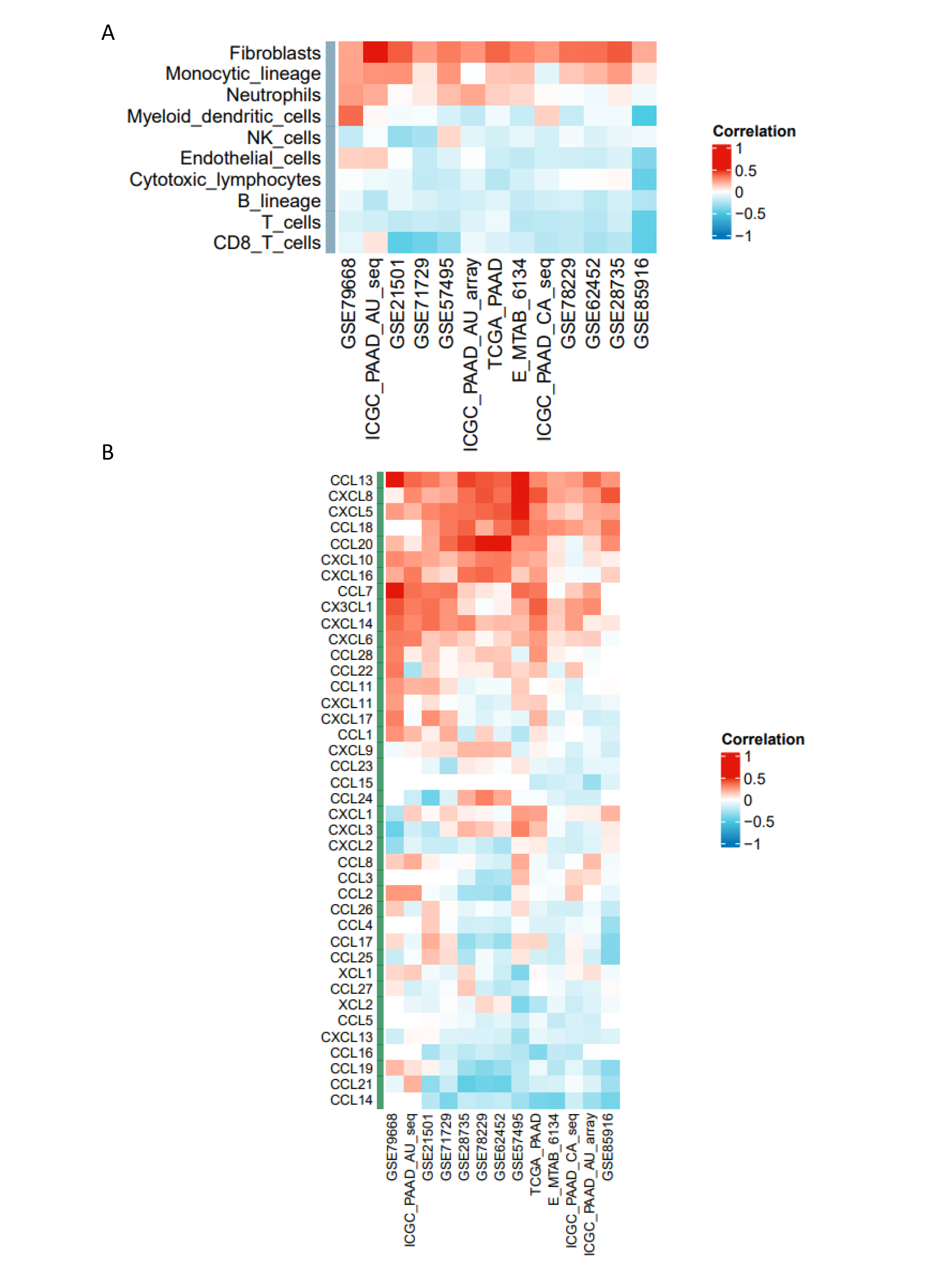

Supplement: Supplementary Figure 4 — The differences in immune cell infiltration and immune factors between the “high C0 score” group and the “low C0 score” group. (A) The immune cell infiltration in the “high C0 score” group and the “low C0 score” group. (B) The correlation between the immune cell infiltration in the “high C0 score” group and the “low C0 score” group and common immune chemokines. [file Image4.tif]
